# Rat Models of Hormone Receptor-Positive Breast Cancer

**DOI:** 10.1007/s10911-024-09566-0

**Published:** 2024-06-24

**Authors:** Raquel Nicotra, Catrin Lutz, Hendrik A. Messal, Jos Jonkers

**Affiliations:** 1 Division of Molecular Pathology, The Netherlands Cancer Institute, Amsterdam, Netherlands; 2 Oncode Institute, Amsterdam, Netherlands

**Keywords:** Rat model, Hormone receptor (HR), Breast cancer (BC), Mammary tumor

## Abstract

Hormone receptor-positive (HR^+^) breast cancer (BC) is the most common type of breast cancer among women worldwide, accounting for 70–80% of all invasive cases. Patients with HR^+^ BC are commonly treated with endocrine therapy, but intrinsic or acquired resistance is a frequent problem, making HR^+^ BC a focal point of intense research. Despite this, the malignancy still lacks adequate in vitro and in vivo models for the study of its initiation and progression as well as response and resistance to endocrine therapy. No mouse models that fully mimic the human disease are available, however rat mammary tumor models pose a promising alternative to overcome this limitation. Compared to mice, rats are more similar to humans in terms of mammary gland architecture, ductal origin of neoplastic lesions and hormone dependency status. Moreover, rats can develop spontaneous or induced mammary tumors that resemble human HR^+^ BC. To date, six different types of rat models of HR^+^ BC have been established. These include the spontaneous, carcinogen-induced, transplantation, hormone-induced, radiation-induced and genetically engineered rat mammary tumor models. Each model has distinct advantages, disadvantages and utility for studying HR^+^ BC. This review provides a comprehensive overview of all published models to date.

## Introduction

Breast cancer (BC) remains the most frequently diagnosed malignancy in women globally, accounting for approximately 12% of all new annual cancer diagnoses [1, 2]. It comprises a heterogeneous disease, with variable clinical outcomes and different histopathological and molecular features [3, 4]. Breast tumors are traditionally classified into distinct molecular groups based on their estrogen receptor (ER), progesterone receptor (PR), and human epidermal growth factor receptor 2 (HER2) expression patterns [5–7]. Such expression levels can be detected by immunohistochemistry, and/or gene expression profiling, yielding three major BC subtypes, namely basal-like, HER2-enriched, and hormone receptor-positive (HR^+^) tumors [3, 6]. HR^+^ tumors, which comprise 70–80% of all invasive cases, are characterized by the expression of ER and/or PR, as well as of many genes expressed by the luminal epithelial cells of the breast, often linked to ER activation [3, 7]. HR^+^ tumors encompass three molecular subtypes, known as luminal A, luminal B, and normal-like BCs. Compared to luminal A BC, luminal B tumors show higher proliferation (Ki67), greater histological grade, decreased differentiation, and a higher frequency of *TP53* and *PIK3CA* mutations [3].

Evaluation of prognosis and treatment options for the different molecular BC subtypes are based on a combination of clinical, pathological, and molecular methods [8, 9]. Along with age, race and menopausal status, an important prognostic and clinical decision-making factor is ER status. Specifically, ER expression in breast tumors is, at least initially, associated with a favorable prognosis, given that patients with ER^+^ BC are more likely to respond to hormonal therapy [10, 11]. However, despite being the recommended treatment approach for ER^+^ BC cases, endocrine therapy still fails to tackle the disease, as more than 50% of patients relapse after > 5 years, frequently with more aggressive and metastatic disease [12, 13]. This can be due to several reasons, including primary or secondary resistance to endocrine treatment, which are still poorly understood [12, 13]. Since HR^+^ tumors represent 70–80% of all BCs, most BC-related mortality can thus be attributed to ER^+^ malignancies and their recurrence patterns [12, 13].

To overcome such setbacks in the clinical management of luminal BC, accurate and appropriate modeling systems recapitulating human disease are pivotal. In the in vitro setting, efforts have been made toward the development of HR^+^ BC cell lines and organoids, although, especially for the latter, loss of HR expression and growth cessation after a few passages remain a challenge [14–18]. Moreover, such models present several drawbacks for the cancer research field, including the lack of a host organism and surrounding stromal and immune cells, and thus failure to recapitulate the tumor microenvironment in vitro [19, 20]. The presence of genetic and epigenetic distinctions between the cell lines and parental tumors following long-term culturing also poses a challenge to the study of disease biology, making these in vitro models unreliable [21, 22]. Such challenges can be overcome by the use of animal models, which not only enable the study of tumor-immune cell interactions, but also the investigation into tumor initiation, progression and metastasis [23].

## Murine Models in Hormone Receptor-Positive Breast Cancer Research

Mice have been instrumental for preclinical in vivo modeling of breast cancer. However, species-specific differences in systemic and mammary gland biology between mice and humans highlight the need for more accurate and representative models in breast cancer research. Compared to mice, rats are more similar to humans in terms of HR signaling and mammary gland architecture. Moreover, rats develop mammary carcinomas that are of ductal origin, HR^+^ and responsive to estrogen, just as the majority of human breast tumors. In the following section we discuss differences between humans, rats and mice, and their relevance for preclinical breast cancer modeling.

### Mouse Models

Mice, due to their small size, short generation times, and genetic modifiability, are frequently chosen for in vivo cancer modeling [24]. In the context of mouse models of HR^+^ BC, transplantation models have been developed based on mouse mammary tumor cell lines allografted in syngeneic mice or on human BC cell lines or patient-derived tumor material xenografted into immunodeficient mice.

**Fig. 1:**
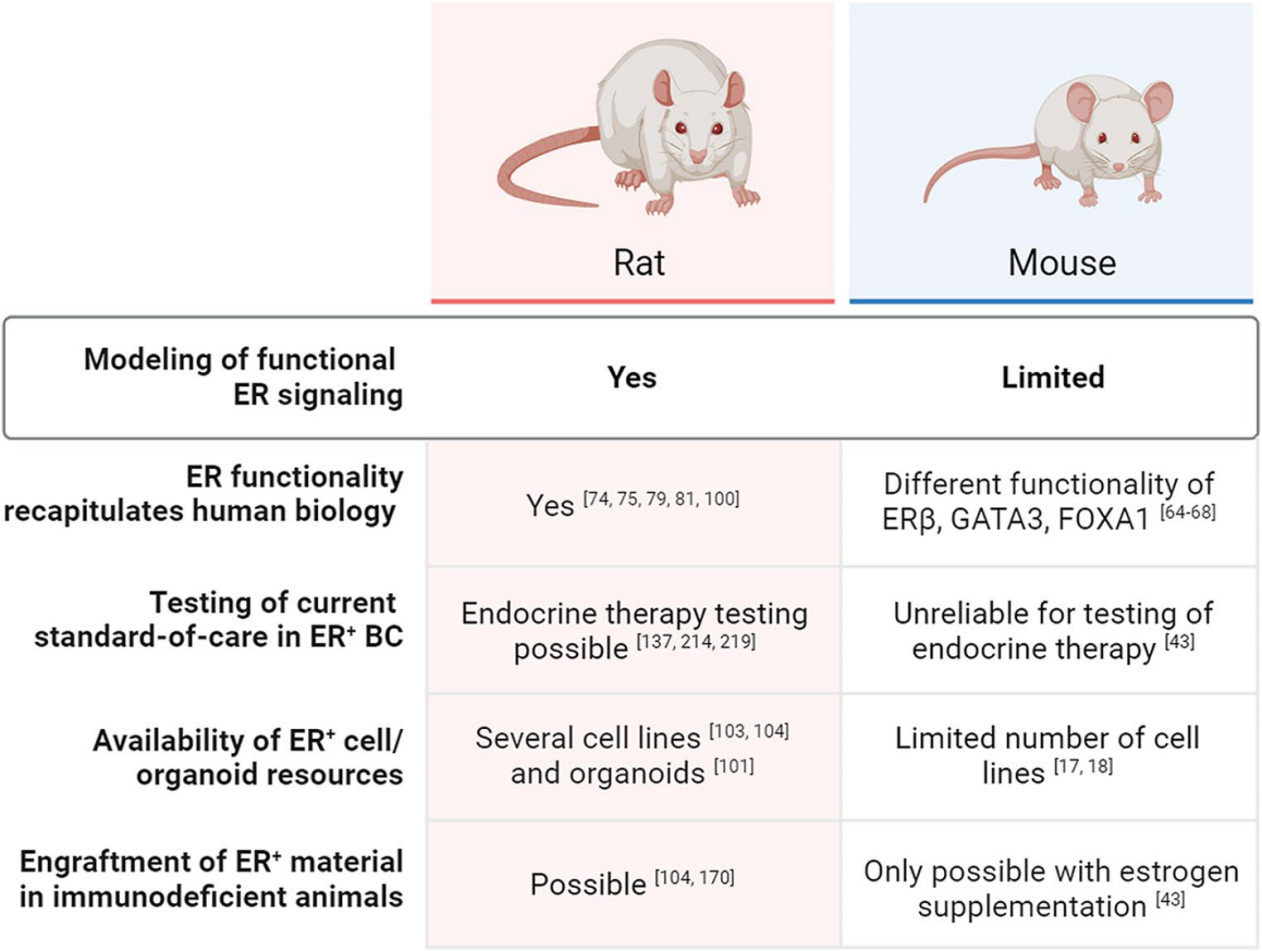
Comparison of rats and mice in the context of HR^+^ BC research, in terms of modeling of functional ER signaling and generated resources

Human BC cell line-derived xenograft (CDX) models present the mainstay of modelling approaches, with MCF-7, T47D and ZR-75-1 being the most commonly engrafted lines [25–29]. Much research has been carried out to compare the molecular characteristics of these cell lines to HR^+^ BC in patients [30–34]. Engraftment of human BC cell lines usually necessitates immunodeficient mice [35], though recent studies have also utilized humanized models [36]. These CDX models have paved the way for molecular and pharmacological studies [28, 29]. Fewer attempts have been made to establish mouse HR^+^ mammary tumor cell lines for allograft studies [17, 18, 37, 38]. Patient-derived tumor xenograft (PDX) models present an elegant alternative to established cell lines, evading the molecular alterations that are known to occur under long-term tissue culture conditions [39]. PDX models are propagated in vivo and recapitulate tumor heterogeneity whilst maintaining patient tumor features over successive generations of in vivo passaging in mice [39, 40]. Large efforts were made to establish a sizable collection of well-characterized BC PDX models with regards to coverage of clinical subtypes, genomic, transcriptomic and proteomic features [41, 42]. Mouse transplantation models, especially PDXs, are still limited by the low engraftment rate of HR^+^ BC, different protein expression profiles compared to the parental tumors [32], and the requirement for supplemental 17β-estradiol (E2) given that endogenous mouse estrogen levels are often unable to sustain xenograft growth [31, 43]. To date, most transplantation models represent triple-negative BC and only few recapitulate HR^+^ BC, and even fewer the luminal A subtype that is most commonly observed in the clinic [44] **(**Fig. 1**)**.

To better recapitulate *de novo* initiation and progression of HR^+^ BC in immunoproficient animals, several strategies have been attempted to establish genetically engineered mouse models (GEMMs) of HR^+^ BC. Initial models mostly relied on the use of the mouse mammary tumor virus (MMTV) promoter, which enabled the direct overexpression of ERα or viral oncoproteins, such as the polyomavirus middle T antigen, in the mouse mammary epithelium [45–48]. However, in these models most mammary tumors are either ER-negative or lose their ER expression at later tumorigenic stages [46, 49–51]. Indeed, the vast majority of GEMMs developed through genetic alterations in estrogen signaling molecules or oncogenes has been reported to predominantly yield mammary tumors that are HR^−^ or lose ER expression when exposed to endocrine therapy [52–54].

To date, only four GEMMs have been reported to develop HR^+^ mammary tumors: the *Stat1*-knockout [55], BLG-*Cre; Kras*^*(G12V)*^ [56], NRL-PRL [57], and Wap-*Cre; Pik3ca*^*(H1047R)*^ [58] models. Though the *Stat1*-knockout, BLG-*Cre; Kras*^*(G12V)*^, and NRL-PRL models develop HR^+^ mammary tumors that are sensitive to hormonal perturbations such as ovariectomy and fulvestrant treatment [55, 56], they all are hindered in their utilization potential by their driver mutations. ER^+^ breast tumors in patients show only low or undetectable levels of STAT1 expression [55], and rarely carry activating mutations in *KRAS* [59], which have been reported to render ER^+^ tumors resistant to endocrine therapy [60–62]. The Wap-*Cre; Pik3ca*^*(H1047R)*^ mouse model overcomes this limitation since *PIK3CA* is commonly mutated in human ER^+^ BCs [63]. Nonetheless, also the Wap-*Cre; Pik3ca*^*(H1047R)*^ model requires continuous E2 supplementation prior to and after tumor onset [58], which contradicts the low physiological levels of E2 in post-menopausal patients [3].

### HR Signaling in Human, Rat and Mouse

ERα signaling is different in mice compared to humans, possibly due to species-specific differences in pioneer factor usage [64]. In addition to the absence of the FOXA1 motif in the mouse Erα binding sites [65], further differences in HR signaling between mice and humans include the lack of Erα36 receptor expression in mice [66], and the distinct roles of growth factor amphiregulin (AREG) and insulin receptor substrates (IRS) in the mouse mammary gland [67, 68]. The lack of Erα36 receptor, a shorter isoform of Erα, responsible for PR regulation in BC [69] and the maintenance of ER^+^ BC progenitor cells [70], has been linked to alterations in post-pubertal mouse mammary duct histology and epithelial cell proliferation [71]. Also, both AREG and IRS are pivotal for mammary epithelial cell proliferation, ductal formation and elongation, and overall mammary gland development in humans [72, 73]. The lack of these factors in mice impairs their alveologenesis and mammary ductal development [67, 68], underlining further differences in mammary gland biology between mice and humans. In contrast to mice, rats have been reported to display HR signaling pathways that are more comparable to those observed in humans and could thus represent a superior model for BC research.

Ovaries and the pituitary gland are two main sources of estrogen production. While the pituitary glands of rats and humans express both ERα and ERβ [74, 75], ERβ expression is absent in the mouse pituitary gland [76]. Given that ERβ overexpression has been shown to enhance estrogen-induced prolactin gene expression [77], its expression in human and rat could indicate that the estrogen regulation of prolactin is likely similar in these species, and distinct from the mouse. Indeed, prolactin signaling has been implicated in promoting ER^+^ tumorigenesis in mice [78] and transgenic overexpression of rat prolactin ligand *rPrl* in the mouse mammary gland induced the formation of ER^+^ mammary tumors [57].

Along the same lines, prolactin-mediated inhibition of lipolysis in adipose tissues is observed in both rats and humans, but not in mice [79], lending further support to the notion that the estrogen-prolactin signaling axis is more strongly conserved between humans and rats, as compared to mice. This is especially relevant, as breast tumor aggressiveness has been linked to the presence of free fatty acids released by the tumor-surrounding adipocytes following lipolysis [80].

Finally, expression patterns of the pioneer factor GATA3 in normal mammary glands and carcinomas are comparable between rats and humans [81], in contrast to the low expression of GATA3 observed in mouse mammary epithelium [65]. Since GATA3 is an essential driver and a prognostic biomarker in ER^+^ BC [82, 83], the similar expression found in human and rat mammary tissues reinforces the relevance of rat models for studying ER^+^ disease and -signaling interactions in vivo **(**Fig. 1**)**.

### Mammary Gland Architecture Differences between Species

Tumors develop in an intricate interplay between cancer cells and their local tissue environment [84]. Mice are characterized by distinct differences in mammary gland architecture compared to humans [85]. Notably, mice do not develop terminal duct lobular units (TDLU) [85, 86], the structure from which luminal tumors typically arise [87, 88]. Furthermore, mice have low levels of serum estradiol, as compared to both humans and rats [89–91]. The latter, coupled with the differences in estrogen signaling and lack of mammary-specific growth factors could, at least in part, explain the architectural, histological and molecular differences observed between human and mouse mammary gland development and tumorigenesis. Indeed, mouse mammary tumors originate from the ductal stem instead of the TLDU and develop with distinct histopathological characteristics, which are more squamous and mesenchymal than those in humans [43, 86], and commonly display gene expression profiles that differ significantly from human lesions [92, 93]. Moreover, mice commonly present with ER^−^ and hormone-independent lesions, in contrast to both humans and rats [64, 94, 95].

**Fig. 2:**
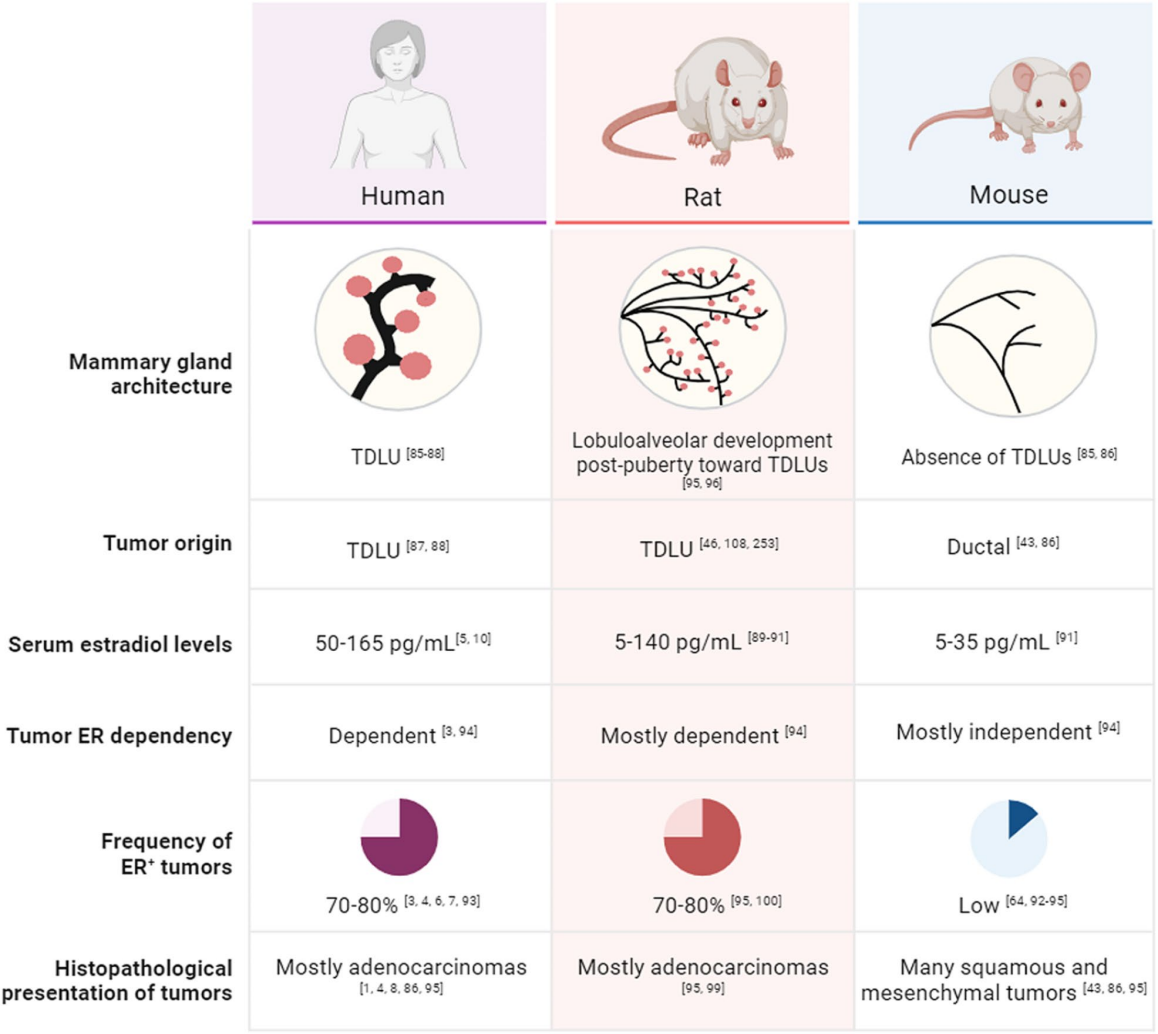
Comparison of human, rat and mouse in regards to their biology, mammary gland architecture and tumor features in the context of HR^+^ BC

Rats possess six pairs of mammary glands that develop lobuloalveolar structures resembling human TDLUs [96]. Compared to mice, drug pharmacokinetics in rats are more analogous to humans [97, 98], and their larger body size facilitates (longitudinal) collection of tumor biopsies and blood, aiding the analysis of drug pharmacokinetics and pharmacodynamics [97–99]. Importantly, unlike most mouse models, rats reliably develop HR^+^ and estrogen-dependent mammary tumors across various models [100] and, thus far, have already enabled the generation of a larger number of ER^+^ mammary tumor cell lines and organoids [101–104] **(**Fig. 2**)**.

**Fig. 3:**
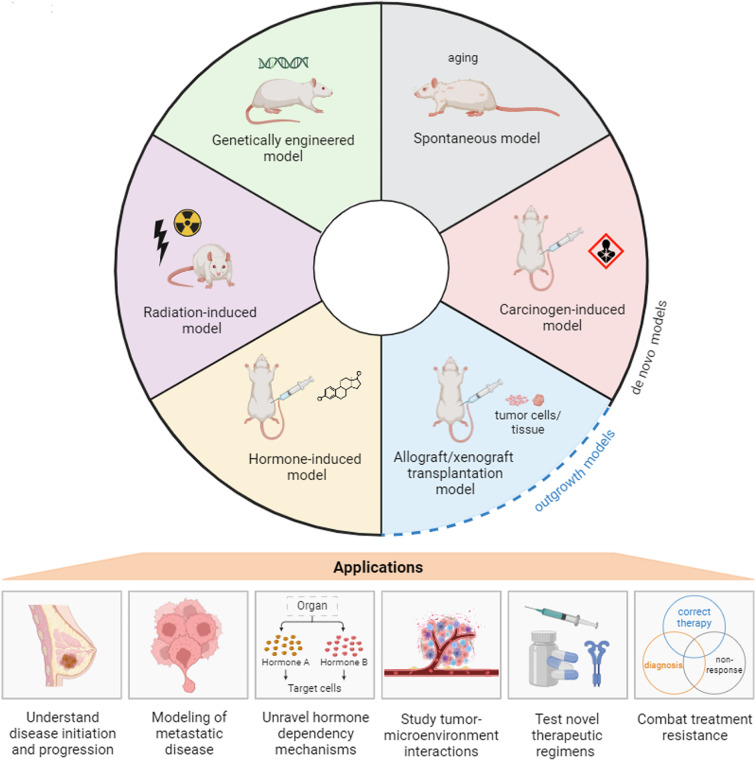
Overview of different rat tumor induction models, clockwise, in order of emergence, and their utility for BC research

**Fig. 4:**
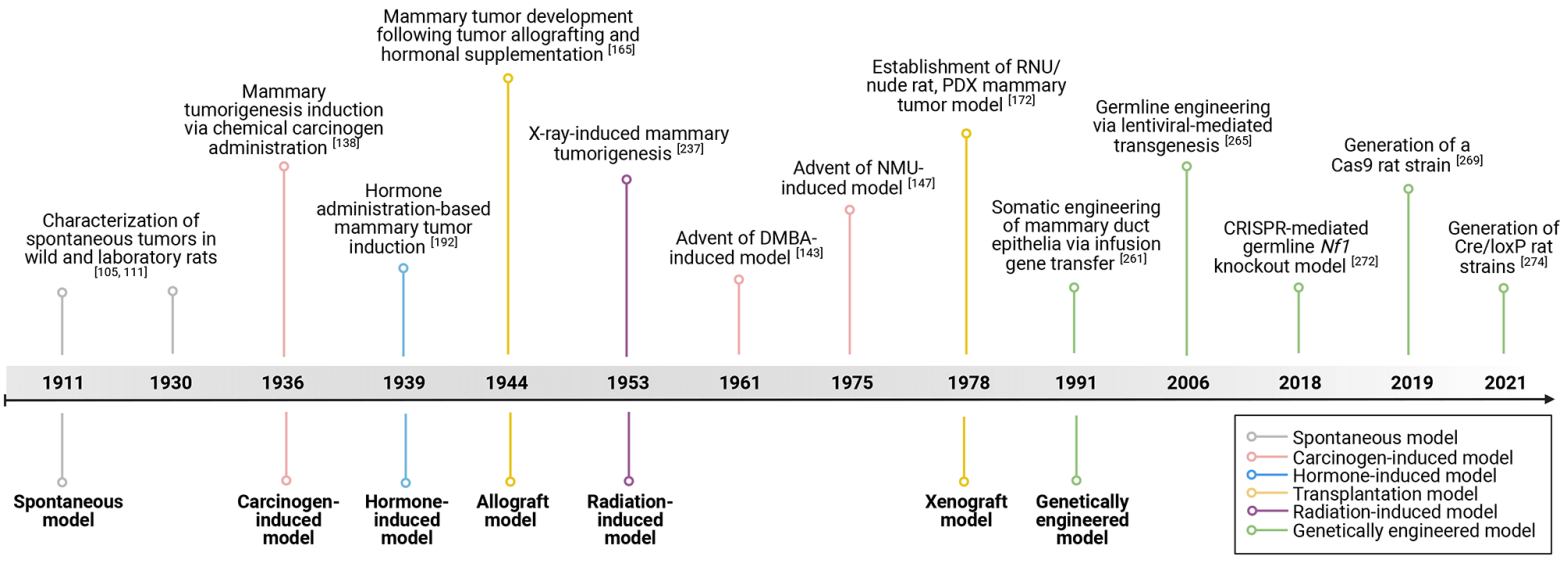
Notable advances in the generation of rat models of HR^+^ BC over the past 113 years. Colors represent the different tumor induction models

Rat mammary tumor models can be categorized into six groups based on their induction methods: spontaneous models, carcinogen-induced models, transplantation models, hormone-induced models, radiation-induced models, and genetically engineered rat models. In this review we discuss, in chronological order, each category of rat mammary tumor models and their (dis)advantages. In addition, we discuss the utility of the various rat mammary tumor models for preclinical studies as well as future research directions in modeling HR^+^ BC in rats **(**Figs. 3 and 4).

## Spontaneous Rat Mammary Tumor Models

### Background

Long before the ability to engineer cancer models, spontaneous mammary tumor systems were the mainstay BC models allowing the study of naturally occurring mammary lesions, which are infrequently observed in experimental animals, including aged female rats of different strains [24, 105–107]. These include Sprague-Dawley (SD), Wistar, August, Albany-Hooded, Copenhagen, Lewis, and Fisher 344 rats, with the latter being particularly susceptible [108, 109]. Since these spontaneous tumors are observed in non-genetically engineered or non-carcinogen treated animals, their development could recapitulate human disease progression and tumor evolution more closely [24, 102, 110]. Historically, the occurrence of these tumors in wild rats has been described since the early 1900’s, by Woolley and Wherry [111], though the first report in laboratory rats was only published in 1930 by Bullock and Curtis [105, 106, 112, 113]. According to the authors, most spontaneous neoplasms in the rat mammary gland give rise to tumors of epithelial origin, of which the majority are benign growths [108, 109, 114].

A similar trend was demonstrated by Bagg and Hagopian [113] and Bryan et al. [112] who, in addition to observing a higher frequency of benign disease in comparison to a lower incidence of malignant growth, shed light on the possible causes behind the neoplastic lesions. These include endocrine disturbances and a decline of the animal’s fertility, alluding to the impact of age-related hormonal changes on mammary tumor incidence [108, 109, 114]. Particularly, the influence of age-associated endocrine perturbations was confirmed in later studies, in which ovariectomy before middle age onset and restoration of estrous cycles in aged animals led to a decrease of spontaneous mammary tumor incidence [115, 116].

In addition to age-related endocrine changes, other factors, such as caloric intake and environmental conditions, were shown to play a role in the development of spontaneous rat mammary tumors [117–119]. Regarding the former, previous studies indicated that, if fed a low-fat diet and presented with a reduction of body weight, rats are likely to exhibit a lower spontaneous mammary tumor incidence, in contrast with those given a high-fat diet and with a higher body weight [117, 120]. Such an effect is also seen in the human setting, with a greater risk of ER^+^ BCs being specifically linked to an elevated dietary fat consumption [120, 121]. Moreover, environmental and housing conditions, such as social isolation, and deregulated light-dark cycles, and thus melatonin levels, have been suggested to influence the occurrence of spontaneous mammary tumors [110, 118, 119]. In patients, similar associations are observed, in particular concerning the cascade effect of sleep deprivation, light exposure at night and diminished melatonin levels in elevated BC incidence [122, 123].

### Advantages

**Fig. 5:**
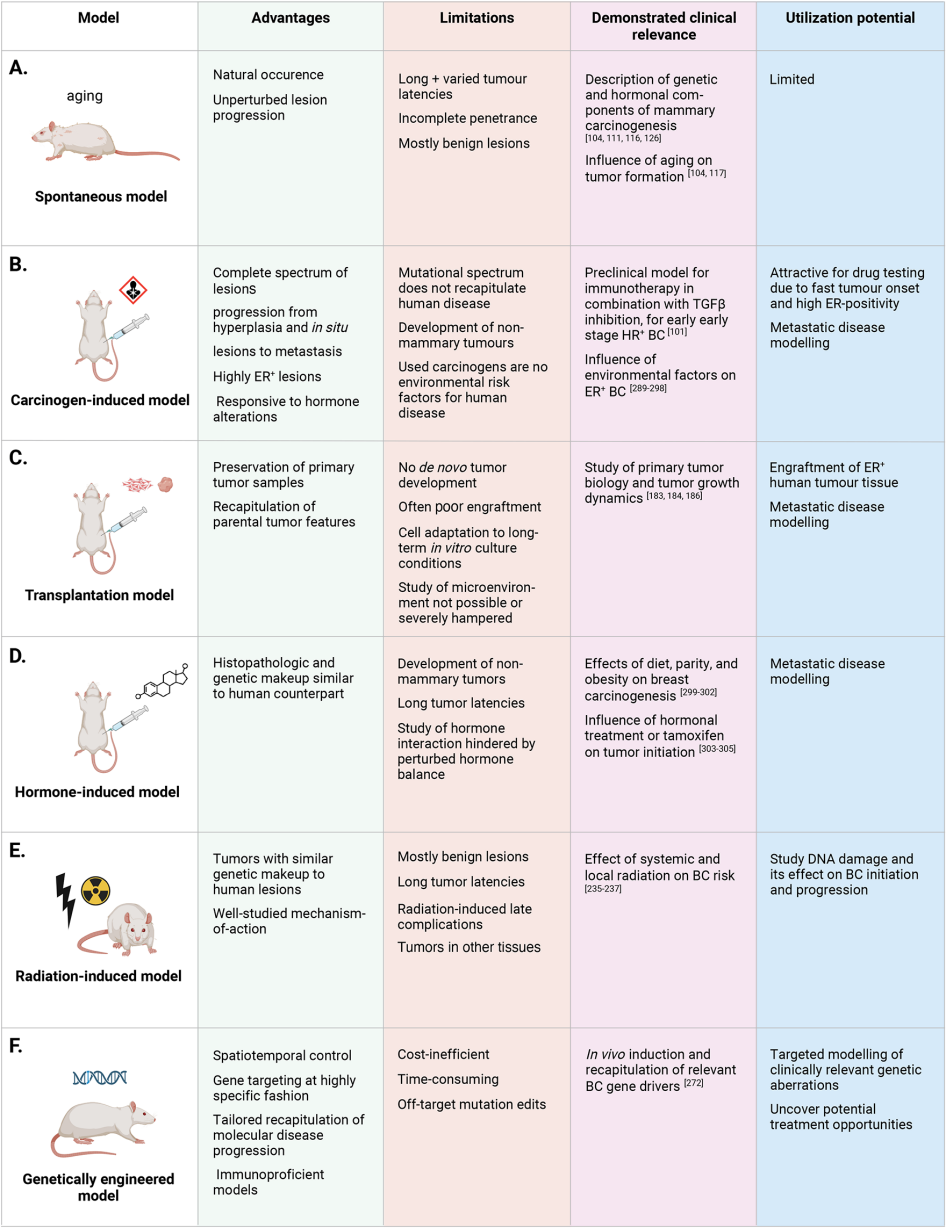
Experimental advantages and limitations as well as unique clinical relevance and utilization potential of the different rat models of HR^+^ BC

One of the main advantages of the spontaneous mammary tumor model concerns their HR expression and dependency status [124] **(**Fig. 5A**)**. As shown by Welsch et al. [125] and Meites [124], the incidence and development of spontaneous tumors is increased in the presence of higher prolactin levels, usually accompanied by a reduction in estrogen secretion. Additionally, previous studies have also shown that hormonal withdrawal, such as ovariectomy and adrenalectomy, or treatment with ER antagonists, can hinder tumor growth and development in SD rats. On the other hand, hormonal supplementation, such as continuous administration of estrogens and prolactin, enhances malignant and benign tumor formation, respectively, highlighting the hormone dependency in such spontaneously occurring tumors [108, 126–128]. Cheung et al. [129] have also shown that, though limited, spontaneous carcinomas in Noble female rats display a positive ERβ cytoplasmic staining, as demonstrated by immunohistochemistry analysis. Another advantage of the model concerns the development of such naturally occurring tumors in genetically heterogeneous populations, similar to the human setting [24].

### Disadvantages

Though in susceptible strains, such as SD and Wistar-related rats, spontaneous mammary tumor incidence is relatively high, most tumors display a benign outgrowth [108, 114] **(**Fig. 5A**)**. These are usually fibroadenomas, fibromas and intraductal papillomas, with malignant disease, such as adenocarcinomas, being a rare and unreliable occurrence [108, 130]. Another disadvantage of this model comprises the long tumor development latencies of up to two years in susceptible strains, rendering it a time-consuming experimental model [24, 108]. Moreover, since these tumors are heterogeneous, and animals display individual tumor growth variations, achieving a statistically significant number of tumor-bearing rats with uniform lesion growth can be challenging. Cha et al. [131] have also revealed that, in untreated conditions, the majority of premenopausal rats display detectable levels of *Hras* mutants, a mutation signature commonly seen in NMU-induced tumors. As subsequently confirmed by McKim et al. [132], such findings highlighted the role of *Hras* activating mutations in spontaneous tumors, deviating from the genetic makeup seen in human BC [131, 133, 134].

## Carcinogen-induced Rat Mammary Tumor Models

### Background

Following the first reports of spontaneous mammary tumors in rats, a second model based on the use of chemical carcinogens emerged. The carcinogen-induced rat mammary tumor model comprises the most heavily used and second oldest preclinical model to study human BC [135, 136]. The system’s generation entails the administration of a single or multiple repeated doses of a chemical carcinogen, usually delivered intravenously or intraperitoneally [137]. Deploying this method, carcinogenic tumorigenesis in the rat mammary gland was first achieved in the 1930s, when Dunning et al. described the development of sarcomas in various rat strains following subcutaneous injection of 1,2,5,6-dibenzanthracene or 3,4-benzopyrene [138]. Subsequently, in 1941 and 1949, Wilson et al. [139] and Shay et al. [140] reported the induction of mammary adenocarcinomas in albino rats following intraperitoneal administrations of the carcinogens 2-acetylaminofluorene (AAF) and methylcholanthrene (MCA), respectively. Their studies were the first to highlight the impact of the endocrine system on rat mammary cancer formation. Specifically, based on their experiments, non-ovariectomized female rats tended to be more susceptible to tumor development, in comparison to males and ovariectomized rats. AAF-induced tumors were also shown to be hindered by lactation, whereas gestation stimulated their growth [141, 142].

Nevertheless, the model’s popularity only came with a study by Charles Huggins, who, in 1961, introduced an improved methodology in which mammary carcinomas in the rat were triggered by a single dose of 7,12-dimethylbenzanthracene (DMBA) [143]. Given its one-dose tumorigenic effects, Huggins’s study enabled closer inspection of the initial and promoting stages of tumor development, alluding to its potential mammary gland ductal origin [142]. Moreover, similarly to other mammary tumor-inducing carcinogens, most DMBA-induced carcinomas are responsive to hormonal changes, as seen by a reduction in tumor growth once animals were ovariectomized, hypophysectomized or an increase in tumor growth once animals received hormone supplementations such as progesterone, estradiol, or a combination thereof [144]. Interestingly, administration of progesterone alone was shown to enhance DMBA-induced tumor growth and incidence, alluding to the hormone’s stimulating effect on tumor development, an event also seen in the clinical setting [144–146].

In light of the successful establishment of a chemically-induced rat mammary adenocarcinoma system in vivo, other carcinogens, such as N-methyl-N-nitrosourea (NMU), were also explored for the same purpose. Gullino et al. [147] were the first to document the induction of primary mammary tumors in different strains of female rats, including SD and Fisher 344, treated with NMU intravenously. In this study, NMU was injected three times, with an interval of four weeks between each dose, yielding a < 70% tumor incidence in all strains tested. All tumors were histologically classified as adenocarcinomas or papillary carcinomas, displayed responsiveness to hormonal alterations via ovariectomy, and were highly metastatic, mainly toward bone marrow and spleen [147].

Given the different published models, the ideal carcinogenic agent and experimental conditions to study human disease were further investigated in follow-up, comparative studies. Gusterson & Williams [148] reported that DMBA and NMU, the most commonly used carcinogens for inducing mammary tumors in rats to date, generate indistinguishable mammary tumors, with both agents failing to trigger local and distant invasion [149]. Gullino et al.‘s claim regarding the NMU-induction tumor model’s highly metastatic potential was also contradicted in Rose et al.'s [150] and Williams et al.'s [151] subsequent work, in which no metastasis was found in the autopsies. In terms of prognostic marker expression, Alvarado et al.’s [149] immunohistochemical analysis comparing tumors from the DMBA and NMU-induced models revealed that, while both groups yielded HR^+^ tumors, NMU-induced lesions presented higher Ki67 and mitotic activity index scores. Such results could suggest that NMU triggers the formation of more aggressive mammary tumors, with similar immunohistochemical characteristics to the luminal B BC subtype [149, 152].

Such differences in the rat mammary tumors induced by DMBA and NMU could be due to their distinct mechanisms of action ​​ [153]. Though both agents rely on DNA alkylation to prompt tumor development, DMBA, unlike NMU, is considered an indirect alkylating agent, as it relies on the metabolic activation by hepatic cytochrome P450 enzymes. As a result, DMBA’s carcinogenic effect is slower, leading to longer tumor latency periods [149, 154]. Moreover, NMU-induced rat tumors were shown to display activating mutations in the *Hras* oncogene in over 85% of cases, whereas *Hras* mutations are rarely found in lesions induced by DMBA [133, 134, 155, 156]. Since *HRAS* mutations are not frequently detected in human BCs, with rates as low as 1%, DMBA-induced rat tumors could thus be a better representation of the human disease [156, 157].

### Advantages

Despite their different metabolic effects on mammary tumor induction, DMBA and NMU share similar cellular targets within the rat mammary gland, namely the epithelial cells of the TDLU, recapitulating the human setting [96, 108, 153]. This reflects the presence of mammary stem and progenitor cells that are prone to malignant tumorigenesis, as well as the TDLUs’ high proliferative and low cell loss profile [137]. When given to rats of susceptible strains at the age of sexual maturity and full mammary gland development, DMBA and NMU can lead to a tumor incidence of up to 100% [100, 136, 137]. These lesions cover a wide range of mammary neoplasms, including benign fibroadenomas, fibromas, and intraductal proliferations. The latter can eventually develop into lesions resembling the human ductal carcinoma in situ, which could potentially develop further to HR^+^ invasive carcinomas [109, 153] **(**Fig. 5B**)**.

Given that over 70% of all invasive human BC cases are HR^+^, the carcinogen-based induction of such tumors in rats offers many advantages to the disease in vivo modeling scene, presently consisting of a negligible amount of mouse models [43]. These include the possibility of exploring new therapeutic strategies for the management of primary BC. Moreover, with the model’s ability to recapitulate the multistep malignant transformation process, including the potential development of invasive lesions from in situ carcinomas, it could also be used to study disease progression [109]. In the same context, the role of the tumor microenvironment in disease development and relapse could also be further explored, as enabled by the use of immunocompetent rats [43].

Furthermore, since this rat model presents hormone-sensitive tumors, fundamental and translational studies could be conducted to further understand the crosstalk between HR signaling and growth factors [43]. For instance, animal modeling-based investigations into the role of the cyclin D1/cyclin-dependent kinase (CDK) 4/6 pathway in BC pathogenesis were essential for the development of CDK 4/6 inhibitors, such as palbociclib [158, 159]. However, similarly to first-line endocrine therapies, intrinsic and acquired resistance to CDK4/6 inhibition still occurs, with over 20% of patients initially failing to respond to the drug, while half of the responding patients relapse within 25 months [160]. This highlights the need for further research on drug resistance mechanisms and therapy efficacy, which could be achieved using the experimental carcinogen-induced tumor model.

### Disadvantages

While the model yields HR^+^ and -dependent mammary tumors, it still presents a few drawbacks in recapitulating BC in vivo. These include the distinct microscopic and macroscopic characteristics of the rat lesions, in comparison to the human counterpart. Specifically, the carcinogen-induced adenocarcinomas in the rat tend to be delineated, with predominant epithelial components. Moreover, rat mammary carcinomas often display a cribriform and papillary aspect with morphologically varying gland-like structures, which tends to differ from the ductal pattern seen in human disease. Another contrasting feature of carcinogen-induced tumors in rats versus human BCs is their low metastasis incidence [161]. This was shown to be the case for both NMU and DMBA-induced lesions, even when they presented multifocally and displayed local aggressiveness [136, 149, 162]. The lack of metastasis in such tumors could be due to the simultaneous and equally proportional proliferation of luminal and basal epithelial cells in the rat mammary gland as suggested by Murad and von Haam [163]. Such proliferative behavior is not seen in the human setting as proliferation takes place mainly in epithelial cells [162]. Furthermore, the DMBA and NMU-induced rat mammary tumor model fails in accurately recapitulating BC in vivo due to the absence of these carcinogens in the human environment and organism [164]. Most importantly, the differing distinct genetic makeup between the human and the NMU-induced rat tumors is a major drawback of the model [133] **(**Fig. 5B**)**.

## Transplantation Rat Mammary Tumor Models

### Background

 The first rat mammary transplantation models were established from carcinogen-induced tumors which were successfully serially transplanted into other recipient rats [165, 166]. A well-documented example of such allograft modeling derived from carcinogen-induced system concerns the 13,762 adenocarcinoma line, obtained from a rat DMBA-induced mammary adenocarcinoma. The tumor line was maintained in vivo through serial passages in syngeneic female rats, as well as cryopreserved stocks [103, 167]. Specifically in Neri et al.’s study [104], tumor pieces were expanded in vivo via subcutaneous implantation into the mammary fat pad of syngeneic female Fisher 344 rats, resulting in histologically similar tumors to the parental lesions. In a follow-up study, a similar trend could be observed when a 13,762 clone was administered as a single cell suspension into the mammary fat pad of syngeneic rats [168]. The generation of this novel, hormone-dependent BC model in rats was particularly relevant for the study of locally recurrent mammary tumors, but also metastasis formation [104, 168].

Paralleling the advent of such carcinogen-derived allografting models, a rat BC xenotransplantation model was developed in 1987 by Vaupel et al. [169], using human tumor specimens. This occurred following the establishment of the RNU/nude rat model in 1978, an autosomal recessive *Foxn1* mutant characterized by a T cell deficiency, while displaying functional B and natura-killer (NK) cells [170–172]. In Vaupel et al.’s study, athymic RNU rats were successfully xenografted with human BC tissues obtained from different patients. Additionally, the RNU strain was deployed for xenotransplantation of small human breast tumor pieces successively passaged in nude mice which were administered via subcutaneous implantations into the flanks of recipient rats, yielding mammary medullary and squamous cell carcinomas [169].

Despite being the only rat strain able to successfully sustain human xenotransplants, RNU rats were lagging behind immunodeficient mice, as the latter were superior in terms of tumor engraftment efficiency [170, 173, 174]. To circumvent this, and in light of the CRISPR-Cas9 technology advances, novel immunocompromised rat models were developed, including the Sprague-Dawley *Rag2/Il2rg* double knockout (SRG) and the *Rag1/Rag2/Il2rg* triple knockout (SD-RG) strains [170, 175, 176]. Such severely immunodeficient inbred rats lacking mature T, B and NK cells have been shown to present not only a 100% engraftment rate of cancer cells, including the human BC cell line HCC1954, but also allow for patient-derived xenograft establishment and expansion [177]. In addition to its human cell line and patient-derived xenografting potential, the SRG rat strain can be applied for the establishment of mouse-to-rat cell xenografts, highlighting the model’s versatility and promising role in the in vivo study of BC development [178].

Lastly, to better recapitulate human disease, immunodeficient rats have been humanized, with the goal of investigating tumor immune microenvironment interactions and immunotherapy responses [179, 180]. In the context of HR^+^ BC, the Rag-/- Gamma chain-/- human signal regulatory protein alpha-positive (RRGS) rat strain, generated by Ménoret et al. [180], represented an important modeling tool for assessing antitumor immune responses in vivo. In this model, *Rag1* and *Il2rg* deficient rats express the human regulatory protein SIRPα on their leukocytes, enabling them to circumvent macrophage-mediated xenograft rejection, leading to the successful engraftment and growth of a patient-derived, ER^+^ and PR^+^ breast carcinoma cell line [179, 180]. Moreover, given the presence of human antitumor immune activities, as well as their inhibition when treated with antibody-based therapy, Ménoret et al.‘s rat model could be further applied for the development of novel therapeutic approaches and regimens for the management of HR^+^ BC.

### Advantages

Though BC initiation and prevention could be investigated with the use of carcinogen-induced models, the understanding of disease progression, especially the in situ to invasive transition stage, was still limited [136, 137]. Such a limitation could be overcome with the advent of transplantation syngeneic rat mammary tumor models, as demonstrated by Chan et al.‘s study [181]. Their allograft BC model was developed by administering a subcutaneous injection of minced NMU-induced rat mammary neoplastic lesions, in suspension, into the mammary gland of the recipient rat, with the originating tumor then being serially transplanted for up to 5 generations. Investigation of tumor development was then enabled via the histopathological analysis of each transplant, highlighting the acquisition of a more invasive phenotype throughout the generations, in contrast to the parental in situ ductal carcinomas [181]. Furthermore, given that syngeneic models derived from carcinogen-induced tumors often generate highly metastatic tumors, as opposed to their carcinogen-induced origin, and similar to the human setting, they could be used to not only track the metastatic process, but also test new anti-metastatic agents [104, 168, 182].

Similarly, PDX and human cell line-derived xenograft rat models, given their direct derivation from human tumor samples, could also represent a valuable resource for drug response testing and understanding of disease progression [24, 183]. Moreover, with the use of immunodeficient rats, PDXs enable BC in vivo modeling with tumor transplants likely to maintain the genomic features of the parental tumor, as seen in PDX BC mouse models [183] **(**Fig. 5C**)**. Alternatively, as a way to further faithfully recapitulate human BCs and their interaction with the immune microenvironment, humanized xenograft models could be applied to not only test immunotherapeutic drugs, but also explore the immune cell infiltration status of HR^+^ tumors [36]. Since most BCs present as immune ‘cold’ tumors, characterized by a lymphocyte-depleted milieu, humanized models could thus enable the investigation into avenues to boost immune cell infiltration in the tumors, consequently leading to immunotherapy responsiveness [36, 184].

### Disadvantages

Despite being a superior species in terms of mammary gland architecture and estrogen-dependent, HR^+^ mammary tumor formation, rats, when immunocompromised, do not perform better than nude mice when undergoing tumor xeno-transplantation [173, 174] **(**Fig. 5C**)**. Specifically, athymic mice have been shown to exhibit a higher tumor establishment success rate, which could be due to a number of reasons [185]. These include the antitumor immune-dependent changes in the rat as it ages, directly affecting tumor growth and transplantability, as well as the ability of regaining immunity with time, which could lead to tumor development cessation [174]. Another disadvantage of cell-line derived transplantation models concerns their adaptation to long term in vitro growth conditions. As a result, genetic aberrations and histopathological differences could arise between the original breast tumor and the derived human cell line, rendering it inaccurate in terms of modeling the human disease setting [35, 183].

Regarding the PDX rat tumor systems, a major limitation of the model is the required use of immunodeficient rat strains, and thus lack of immune cells in the tumor microenvironment. This hinders the study of natural human tumor development and perpetuation in the in vivo system, and potential application of immunotherapy for HR^+^ tumors [183, 186]. Additionally, engrafted cancer cells in PDX models tend to undergo clonal evolution within the tumors, heterogeneity loss, and a possible selection bias for transplantation [187, 188]. Humanized xenograft rat models, an improved alternative to immunocompromised ones, could also display a number of drawbacks, including the limited development of mature immune cells and the onset of graft-versus-host-disease, which could be lethal to the recipient animal as observed in mice [189].

## Hormone-induced Rat Mammary Tumor Models

### Background

In addition to playing a pivotal role in the establishment and characterization of the carcinogen-induced rat mammary tumor model, Geschickter was the first to describe rat mammary carcinogenesis following hormonal treatment [190, 191]. Their work revealed that the injection of different estrogenic substances, including estrone (E1), estradiol and diethylstilbestrol, yield cancer formation as early as 25 days post-dosage. Most importantly, Geschickter’s study highlighted that estrogen-induced rat mammary carcinomas are a result of physiological changes triggered by the hormones, rather than their carcinogenic nature [190, 191]. This is in line with epidemiological and experimental evidence on the role of hormonal imbalance in BC development [192]. Specifically, previous studies have demonstrated a link between higher BC incidence and prolonged exposure to estrogen due to, for instance, late menopause, obesity and long-term hormone replacement therapy [193–195]. Such estrogen-related breast tumor initiation and progression is dependent on both ER-dependent and –independent mechanisms, which are responsible for the expression of ER-responsive genes, cell proliferation, as well as the production of tumorigenic, DNA-damaging estrogen metabolites [196].

Regarding its administration mode, and similarly to what has been described in Geschickter’s protocol, estrogen induced mammary tumor development in rats can be promoted via subcutaneous pellet, silastic implants, or repeated intramuscular injections of the chemical dissolved in oil [197–200]. Among the different estrogen variations, the naturally occurring form, namely E2, is the most frequently applied, though diethylstilbestrol, estrone and 17α-ethinylestradiol have also been used [137, 201]. In addition to estrogen, other hormones, such as testosterone and progesterone, were shown to contribute to breast carcinogenesis, and have thus been used to model the disease in vivo [201, 202]. Interestingly, the administration of repeated progesterone doses to SD rats previously treated with MCA or DMBA resulted in enhanced mammary tumor formation, suggesting a synergistic effect of the combination of the two chemicals [198, 203, 204]. Combinations of E2 with progesterone and testosterone resulted in higher mammary tumor incidence, in contrast to either hormone alone [201, 205, 206].

To obtain such results, two different susceptible strains have mainly been used, namely the Noble (Nb) and August–Copenhagen–Irish (ACI) rats, two well-known models for hormone-inducible rat mammary carcinomas [201]. First described in the early 1940’s, by Noble et al., the Noble rat model enabled a better understanding of mammary tumor progression in the presence or absence of a hormonal stimulus [207, 208]. Their work was also crucial for the examination of the molecular mechanisms underlying hormone-induced mammary tumorigenesis, highlighting the role of different oncogenes, such as *Ccnd1* and *IgF2*, in tumor progression [209]. Similarly, the ACI rat model, initially established in 1997, is another unique rodent model that gained notoriety for being able to display a 100% mammary tumor incidence in the presence of continuous E2 supplementation at physiological levels. These are similar to those observed during the human periovulatory phase of the menstrual cycle or pregnancy, underlying the relevance of E2 levels for elevated disease incidence observed in the human setting [200, 201].

### Advantages

The establishment of the Noble and ACI rat hormone-induced mammary carcinoma models offers a number of advantages in the BC research field, including the study of early tumor formation following hormonal treatment. As shown by Mense et al. [196], susceptible ACI rats can display mammary hyperplastic lobular units within 7 days of E2 exposure, with subsequent hyperplasia and ductal elongation within 15 days of treatment. As hormonal administration continues, luminal epithelial proliferative responses are triggered, eventually leading to the formation of ductal carcinomas in situ and invasive mammary carcinomas [201]. Though with a longer latency time, a similar tumorigenesis trend can be seen in the Noble rat model described by Xie et al. [205], with carcinoma lesions being fully developed after 5 to 6 months post hormonal treatment onset. Most importantly, such hormone-induced mammary carcinomas have been shown to express PR, ERα, ERβ, as well as the GATA binding protein 3, a transcription factor involved in the mammary luminal epithelium development [129, 201, 210]. Such molecular features are also present in human luminal BC, suggesting that rat mammary tumors induced in this model are not only hormone-sensitive, but also resemble the human setting [201] **(**Fig. 5D**)**.

In addition to the expression of luminal BC markers, hormone-induced rat mammary tumors display similar genetic alterations to the ones observed in human disease [129]. Specifically, Li et al. [211] have indicated that E2-induced mammary tumors in ACI rats, similarly to invasive human ductal BCs, exhibit high degrees of c-MYC overexpression and amplification and genome instability. The latter comprises high levels of aneuploidy, accompanied by nonrandom gain or loss patterns of specific chromosomes, a characteristic seen in approximately 85% of BCs [201, 212, 213]. Moreover, multiple quantitative trait locus analysis of the ACI rats in comparison to non-susceptible strains underlined the existence of estrogen-induced mammary cancer (*Emca*) loci, which foster genetic determinants of E2-induced mammary tumor susceptibility. Notably, such *Emca* loci are orthologous to the genetic determinants of BC risk in humans, as demonstrated by genome wide association studies [214–216]. Altogether, these data highlight the genetic simi​​larities between the lesions in the different species, thus enabling potential functional studies on hormone-dependent BC risk and incidence [216].

Another advantage of hormone-induced rat mammary tumor models concerns the possibility of impelling tumor growth regression via hormone administration cessation or in the presence of selective ER modulator drugs, such as tamoxifen [137, 212, 217]. As demonstrated by Harvell et al. [212], and unlike other rat mammary tumor models, E2-induced tumors in ACI rats completely regress following E2 implant removal, indicating their dependency on exogenous E2. The same could be observed in Noble and Collip’s [208] study which described tumor growth recession also in the absence of progesterone. Their experiments also demonstrated that the novel hormone-induced tumor can appear and grow in a continuous manner [208]. The in vivo visualization of hormone-dependent tumor growth was essential for the development of anti-estrogen drugs, eventually leading to the production and optimization of Fulvestrant, the first selective ER down-regulator (SERD) currently used as standard-of-care for HR^+^ BCs patients [218–220].

### Disadvantages

Despite its many advantages for the study of HR^+^ BC in vivo, the use of hormone-induced rat mammary tumor models also presents drawbacks. These include the possible formation of primary lesions in hormone-independent organs, and the influence of the animal’s age on the tumor latency [129, 137] **(**Fig. 5D**)**. With regards to the former, previous studies on the carcinogenic role of endogenous estrogens E1 and E2 and their products in two different rodent species revealed that tumor induction also takes place in the kidney, where the genesis of human hormonal cancers does not typically occur [221–223]. Such an event could be explained by the presence of specific mutations that lead to abnormal cell proliferation and cancer formation. In the case of diethylstilbestrol, these mutations arise from the product of a complex interaction between the hormone’s catechol quinones and DNA, rather than estrogen-receptor mediated cell proliferation reactions [222–224]. Thus, hormone-induced rat mammary tumors might be derived from the genotoxic effects of E1/E2 quinone metabolites, previously shown to trigger mutations responsible for initiating various human cancers [225].

Concerning the role of the animal’s age on mammary tumor development, Geschickter and Byrnes [226] have previously demonstrated that younger rats on estrogen supplementation tend to present a longer tumor latency period, in comparison to older animals. For instance, one-month old rats displayed a tumor latency of 42 weeks, whereas 20-month old animals exhibited a latency of 13 weeks, indicating a possible protective effect of younger age against estrogen inducible mammary carcinogenesis [137, 226]. Given that older female rats tend to develop spontaneous mammary tumors, with their incidence proportionally rising as age increases, tumor development initially considered to be hormone-induced, could instead be attributed to animal age [102, 107]. Though this can be counteracted with earlier experimental time points, the period for tumor latency and maintenance of physiological hormone levels, when giving continuous hormonal supplementation, can be at cost.

## Radiation-induced Rat Mammary Tumor Models

### Background

Ionizing radiation exposure has long been established as one of the environmental, etiological factors of human BC, with lesions documented following doses as low as 0.1 to 0.5 Sv [227–230]. While most knowledge on the impact of radiation on BC incidence is derived from epidemiological data on atomic bomb survivors and patients exposed to diagnostic or therapeutic radiation, in vivo experimental models played a major role in improved understanding of radiation-induced breast carcinogenesis [229, 231, 232]. In particular, radiation-induced rat mammary tumor models were crucial for the study of dose-response relationships and the molecular mechanisms underlying tumor formation in mammary tissue [233, 234]. Following the publication of the first radiation-induced rat mammary tumor model in 1953, in which tumor development was induced in X-ray irradiated Sprague-Dawley rats, several other strains, radioactive agents, dosing and exposure time points have been tested, as well as combinatory studies of radiation- and hormone- or chemical-induced mammary tumor carcinogenesis [230, 231, 235–237].

Concerning the different radioactive carcinogens used to induce mammary tumors, sparsely ionizing, including X and γ-ray, and densely ionizing, such as neutron and carbon ions, have been used, though γ-radiation is the most prevalent type [238–242]. Rats can be exposed to such agents via a single radiation dose, systemically delivered to the entire body, or, though less frequently performed, a local dose, administered to a specific body part [108, 233]. Regardless of the radiological agent and administration mode, irradiated rats appear to undergo a similar somatic mutational reaction to the radiation, leading to the development of corresponding mammary tumor identities [239, 242]. Such tumors have been shown to develop within approximately 140 days to up to one year succeeding radiation exposure, with neoplasm incidence being directly proportional to the dose given [137, 243].

Radiation-induced mammary carcinogenesis in the rat is a result of two main molecular events, namely DNA damage, through double strand breaks, and the generation of reactive oxygen and nitrogen species, both triggered by DNA and protein oxidation [137, 244]. DNA damage consequently leads to mutations, copy number losses, deletions, chromosomal amplification, and an overall increased genomic instability, making cells prone to tumorigenesis [137]. Specifically, Loree et al.‘s [234] study on irradiated rat mammary tissue has indicated genome-wide hypomethylation, accompanied by down-regulation of the expression of DNA methyltransferases. Furthermore, their work highlighted alterations in cellular proliferation, apoptosis, and pro-survival signaling post-irradiation, with expression of cyclins D1 and D2, two known carcinogenesis markers, being notably elevated [234].

Among the different rat models used to study radiation-induced carcinogenic effects, previous studies have demonstrated tumorigenesis susceptibility in at least 4 different strains, namely the Sprague-Dawley (SD), Wistar-related, including Wistar Albino Glaxo and Lewis, Copenhagen, and Long-Evans (LE) rats [231, 233, 235, 243, 245, 246]. In particular, SD rats demonstrate elevated sensitivity to radiation, also at low doses, linked to a high incidence of mammary neoplasms [245, 247]. A similar tumor burden trend was also observed in the LE strain which, like SD, scored a 56% tumor formation rate, in comparison to 5% in Wistar-Lewis [245]. On the other hand, Shellabarger [248] has shown that the Lewis strain yields mammary adenocarcinomas in a comparable rate to SD rats, while not displaying a mammary fibroadenoma response, making it a valuable model for the study of adenocarcinoma formation. Moreover, rats of the Fischer F344 strain, when previously implanted with estrogen pellets, have been shown to respond to X-ray exposure, yielding mammary carcinomas at a high incidence [249, 250].

### Advantages

In addition to being a well-studied tumor inducer that elevates the risk of BC formation in the human setting, ionizing radiation enables modeling of HR^+^ adenocarcinomas in rats. These tumors tend to display similar genetic alterations to those previously reported in human BCs, thus representing a relevant model to the study of luminal BCs in vivo [244] **(**Fig. 5E**)**. Specifically, a study performed by Moriyama et al. [242] revealed that both neutron and γ-radiation exposure results in an increased incidence of luminal mammary adenocarcinomas in SD rats, positive for ER and/or PR, while negative for HER2 in comparison to the non-irradiated control. Their study has also proposed the presence of focal copy-number losses in certain genes, including the tumor suppressor gene *Cdkn2a*, as a signature of radiation-induced rat mammary tumors [242]. Moreover, radiation exposure is shown to target mammary cells within the TDLU, resulting in a number of cellular alterations up to 8 weeks post-irradiation [251]. Early changes included persistent proliferation of TDLU cells, followed by the development of preneoplastic lesions, resembling the oncogenic process seen in human BC [96, 251].

### Disadvantages

The main disadvantage of radiation-based treatments is the high incidence of benign lesion growth, such as adenomas and fibroadenomas and the long latency periods [230]. In addition, and especially at high radiation doses, the model is hindered by the development of late complications, including vascular injury, formation of fibrotic tissue, necrosis and atrophy. In BC patients, radiation cardiotoxicity is a common side effect, as well as the formation of secondary malignancies, such as acute leukemias [252] **(**Fig. 5E**)**. In fact, the presence of radiation-induced myeloid leukemia in laboratory mice has been observed since the 1930’s, with its incidence increasing with age at which radiation exposure occurs [253, 254]. Though the same has not been documented in rat models thus far, Huggins and Fukunishi [255] observed the occurrence of osteosarcomas, as well as of mesentery and intramuscular sarcomas in SD rats post-irradiation. Such off-target carcinogenic effects could hinder proper mammary tumor disease modeling, as the animals’ movement could become compromised at an early time point [256]. Furthermore, given that animals are typically exposed to whole-body irradiation, their overall wellness and natural behavioral patterns might be compromised, leading to ethically deviant experimentation settings [257].

## Genetically Engineered Rat Mammary Tumor Models

### Background

Unlike mouse models, in which genetic strategies have been widely used for various genome manipulations and functional studies, genetically engineered rat models for BC are still rather scarce [258]. Genetically manipulated models can be classified based on the type of mutations being induced, namely somatic and germline mutations. The first somatically engineered rat mammary tumor model dates back to 1991, entailing the administration of transgenic constructs containing a v-HA-ras-expressing viral vector into the mammary ductal epithelium, giving rise to the infusion gene transfer model [259]. This model’s administration is equivalent to the mouse mammary intraductal (MIND) method, initially used to mimic progression of ductal carcinoma in situ lesions in vivo, using cell lines or patient-derived tissue [260, 261]. Such a tool enables specific anatomical targeting with the retroviral constructs, which only incorporate into the genome of proliferating mammary ductal epithelial cells [258]. In addition to retroviruses, lentivirus and adeno-associated virus (AAV) vectors can be used to deliver genetic content and trigger in situ genome editing in rats, as done in mouse models [100, 262].

With respect to germline models, lentiviral transgenic technology in rats has been used, as highlighted by Dann et al., to generate new models with a stable and inheritable phenotype following depletion in a gene-targeted fashion [263]. The method enables targeted in vivo gene knockdowns through RNAi, and the successful delivery of shRNA-based vectors by lentiviruses [263, 264]. Prior efforts to that were hampered by the low efficacy and embryo survival in rats following pronuclear microinjection of a transgene into a fertilized oocyte, a technique typically used in transgenic mouse line production, and by the lack of easily maintained rat embryonic stem (ES) cells, which could be genetically manipulated prior to host implantation [100, 258, 265]. The latter, an issue subsequently amended by improved rat ES cell derivation and expansion, is particularly relevant for the field given the method’s ability to generate knock-ins and conditional and inducible knockouts [264].

Moreover, in vitro genetic manipulation of spermatogonial cells significantly improved with the advent of the CRISPR/Cas9 technology, leading to targeted germline mutations in rats, and opening doors to novel rat knockout transgenic models targeting different genes of interest [100, 266, 267]. The CRISPR-Cas9 genome editing system has reshaped the mouse cancer BC modeling field by enabling somatic indel manipulation of tumor suppressor genes and missense mutations in proto-oncogenes [268, 269]. Specifically for rat HR^+^ mammary tumors, Dischinger et al. [270] have demonstrated that the CRISPR-mediated germline knockout of *Nf1*, a regulatory gene in the RAS pathway linked to increased luminal BC risk, leads to estrogen-dependent ER^+^ mammary tumors in SD rats [270, 271]. Dischinger et al.’s model is the first to describe the successful generation of germline knockout models using a CRISPR/sgRNA design injected into the pronuclei of fertilized rat zygotes.

Though no BC rat models with both germline and somatic engineering conditions have been established to date, the generation of a Cas9-tolerant rat strain, characterized by a Cre-recombinase dependent, CAG-promoter driven expression of Cas9 in the *Rosa26* locus, could offer new opportunities in the genetically engineered rat modeling scene [267]. The establishment of such a germline Cas9-tolerant rat model could be applied to somatically model and study loss-of-function mutations. Along the same lines, the recent establishment of a Cre-rat resource, which includes 10 tissue-specific, inducible Cre-rat lines, deploying the Cre-ERT2/loxP system, could enable the further exploitation of the model for the study of HR^+^ BC [272].

### Advantages

Genetically engineered rat mammary tumor models, though still limited in numbers, present several advantages for the HR^+^ BC research field. These include the possibility of directly targeting the ductal structures of the rat mammary gland via, for instance, somatic engineering through the MIND methodology, and thus only genetically modifying the epithelial cells from which BC arise, and the ability to control timing of tumor initiation [259, 260, 273, 274] **(**Fig. 5F**)**. The latter is especially relevant for viral-based genetic engineering systems, presenting unique opportunities, such as long-term and stable transgene expression, low immunogenicity and the ability to sustain inserts of up to 3500 bp [275, 276]. Moreover, given that the spread of these viruses is hampered by a defect in virus replication and the lack of a helper virus, it is possible to regulate the frequency of modified cells and viral integration through virus titration or hormonal stimulation of the mammary gland, respectively [259].

Gene editing through homologous recombination in rat-derived ES cells could also play an important role in the BC rat modeling field, as previous studies have revealed the method’s suitability in achieving precise genetic modifications, including gene replacements and chromosomal rearrangements, which can lead to novel knockout models via germline transmission [264, 277]. Such gene knockouts could also be induced or conditioned when combined with Cre/loxP systems, thus enabling temporal control and tissue targeted alterations in tumor suppressor genes [277]. Additionally, the combination of ES cells and Cas9-mediated gene editing has been shown to be a highly efficient genetic tool for the generation of compound gene mutant models [278]. This could be achieved by targeting various genes in one rat embryo via one RNA microinjection, as demonstrated by the authors. Though not yet applied for HR^+^ BC rat modeling specifically, further optimization of such methods in rats could provide a novel and potent platform for the study of human disease [277].

Moreover, genetically engineered models enable the targeted disruption of genes, and thus the possibility of recapitulating the disease genetic loci, and induction of specific overexpression, knockout or mutations in cells and tissues of interest [279–282]. As shown in Cas9-based base editor (BE) and prime editor (PE) mouse models, precise gene edits can also be achieved through somatic engineering [281–283]. Most importantly, and in light of the advent of immunotherapy, genetically engineered models are immunocompetent, thus making them valuable resources to the study of novel immunotherapeutic approaches, as well as the effects of certain genetic modifications on the tumor microenvironment [265].

### Disadvantages

Despite its many advantages and possibilities for the BC in vivo modeling field, rat genetically engineered models also exhibit limitations. Viral-based methods are often limited by the vector packaging capacity, the inability to modify several genes simultaneously, and the difficulty in attaining full gene ablation [263, 264, 284]. One of the major disadvantages of ES cell-mediated gene targeting is the establishment of germline-competent rat ES cell lines, as cell line injections into recipient blastocysts are required to generate chimeric animals. These are then extensively bred with the purpose of producing offspring with the manipulated ES cell genetic make-up. As a result, the method is considered to be laborious, expensive and time consuming [285] **(**Fig. 5F**)**. In the case of CRISPR-Cas9 gene editing systems, off-target mutation edits could take place, as previously seen in cell lines and mouse models [281, 286]. This could similarly hinder the establishment of somatic modeling in rats. 

## Applications

The wide spectrum of rat BC models has been crucial for understanding BC initiation, progression, metastatic disease, has shed light on risk factors, and enabled testing of various pharmacological compounds, including hormonal therapies. The development of genetically engineered rat models will enable tailoring therapies to patient groups with specific mutational signatures, moving toward more personalized treatment avenues **(**Fig. 5A-F**)**.

### Resource Generation

Spontaneous mammary tumor models, given their naturally occurring incidence, have limited research applications but have been pivotal for developing rat tumor cell lines and studying the genetic and hormonal components of rat mammary carcinogenesis [102, 103, 246] **(**Fig. 5A**)**. Notably, carcinoma cell lines derived from spontaneous tumors were characterized as being ER^+^ and estrogen-dependent [102], demonstrating transplantability and retention of parental tumor growth rates and histological features upon engraftment into immunodeficient mice [23]. Following those pioneering studies, the advancement towards induced BC models has enabled further insights into BC risk factors, mechanisms of progression, and potential treatment opportunities.

### Elucidating Breast Cancer Risk Factors

Risk factors of cancer progression have mainly been studied using rat models relying on carcinogens, hormones or radiation. DMBA– and NMU-induced rat mammary tumor models have significantly contributed to understanding tumor-modulating environmental factors [287–294] **(**Fig. 5B**)**. A carcinogen-induced model was also successfully deployed to show that weight gain prevention after menopause reduces the risk of obesity-associated tumor development [295]. Also, the hormone-induced rat mammary cancer model has been instrumental in diet-gene interaction studies, pharmacological research on endocrine therapies, as well as the exploration of food antioxidants [217, 296, 297]. For example, the hormone-induced ACI rat model has been crucial in investigating energy restriction diets and the effects of vitamin E supplementation on preventing and managing E2-induced mammary lesions [297, 298]. Along those lines, studies employing radiation-induced BC models highlighted that parity and age of radiation exposure [299], and elevated insulin and leptin levels, leading to increased energy availability, promote mammary tumor development [300]. Furthermore, the ACI rat model allowed for evaluating the effects of tamoxifen on E2-metabolism mediated ROS production and DNA damage [217, 301] **(**Fig. 5D**)**. Peterson et al. [302], using a radiation-induced BC model, found that rat carcinomas driven by the Her2/Neu pathway are more prone to tamoxifen chemoprevention failure, demonstrating a need for alternative therapeutic strategies.

### Breast Cancer Initiation and Progression

Mechanisms of cancer initiation have first been studied using the carcinogen NMU, offering initial insights into disease progression and the transition from in situ to invasive disease [136, 137]. Following this, syngeneic animals generated from this carcinogen-induced model have shown to produce highly metastatic BCs, mimicking the human scenario and allowing the study of metastasis, a phenomenon rarely captured by other models [104, 168, 182]. In addition to Gullino et al.’s [147] observation of metastatic lesions to the bone marrow and spleens of rats with NMU-induced mammary tumors, lung and lymph node metastases have been reported in estrogen-induced mammary tumor [191] and transplantation models [104], highlighting the value of rat BC models for the study of metastatic disease **(**Fig. 5B-D**)**.

The advent of immunodeficient rat strains [175], together with naturally higher estrogen levels in rats, present an opportunity to establish PDX biobanks as previously done in mice, with the advantage to model ER^+^ BC without exogenous hormone supplementation **(**Fig. 5C**)**. Genetic engineering has further paved the way for models recapitulating specific molecular BC subtypes and mutational signatures, and for studying the impact of specific germline or somatic mutations in tumor suppressor genes or oncogenes associated with BC [262, 270, 303, 304], such as the creation of rats with germline *Nf1* mutations using CRISPR/Cas9 gene editing [270] **(**Fig. 5F**)**.

### Development and Testing of Breast Cancer Treatment Strategies

Historically, clinical BC therapies have been developed using some of the earliest established rat models. DMBA-induced tumor models have facilitated pharmacological studies testing a range of drugs, such as letrozole, palbocyclib, lapatinib, tenofovir alone or in combination with doxorubicin, and sodium channel inhibitors [158, 159, 305–307]. Furthermore, NMU-induced models have been used to study BC prevention, [308] as well as for nutritional and pharmacological studies focusing on hormone-related effects, [309–311] and as a preclinical validation system for immunotherapy responses [101]. Importantly, allografting NMU-induced tumor pieces from inbred rats, such as Fischer 344, into syngeneic recipients has expanded the use of these models to test new anti-metastatic agents [104, 168, 182] **(**Fig. 5B**)**.

### Tumor Immunology Studies

Whilst the rat immune system remains to be fully elucidated, it bears striking similarities with the human counterpart [312]. Rats may therefore present an opportunity to disentangle tumor-immune interactions and uncover immunotherapy treatment avenues in HR^+^ BC. In fact, several studies have broken ground to combine rat modelling and tumor immunology. Rat mammary adenocarcinoma cell lines derived from carcinogen-induced tumors from inbred Fischer 344 rats were used to study the role of the immune system in tumor development and progression in rats [313–315]. These studies elucidated NK cell activity against the MADB106 rat adenocarcinoma cell line transplanted in syngeneic rats, highlighting the tumoricidal interaction between NK cells and tumor cells. More recently, a study by Gil Del Alcazar et al. [101] has shed light on the role of the mammary tumor microenvironment in immune escape and responsiveness to immunotherapy using the NMU-induced rat mammary tumor model. These NMU-induced tumors displayed an evolution pattern within their microenvironment similar to the immune selection and editing that occurs in human cancers, rendering the model a useful platform to study tumor-immune interactions in vivo [101] **(**Fig. 5B**)**.

## Discussion & Future Perspectives

Animal models have been shown to play a major role in the biological understanding of BC, enabling disease monitoring and the study of cancer initiation and progression in vivo [24]. In the context of HR^+^ BC, the similarities between rat and human mammary tumorigenesis have made these rodents a promising species to model the disease, overcoming some of the challenges of modeling such tumors in mice [43, 137]. Particularly, in contrast to mice, rats display TDLU-like structures as they are found in human breast anatomy, recapitulating the architectural makeup of the human compartment most commonly originating breast malignancies [87, 88, 96]. In addition to yielding tumors of ductal origin, rat models have been shown to develop HR^+^ and estrogen-dependent mammary tumors, with similar histopathologic characteristics to the human lesions [99, 109]. Such features make rats useful models with potential for even greater utility to study HR^+^ BC in vivo, as demonstrated by several modeling efforts and techniques reported to date.

Based on the tumor induction method, six distinct categories of rat mammary tumor models have been established, namely the spontaneous, carcinogen-induced, transplantation, hormone-induced, radiation-induced, and genetically engineered models. Though each model is based on distinct induction agents and carcinogenesis mechanisms, they have all been shown to induce HR^+^ mammary tumors [124, 147, 181, 201, 242, 270]. However, given that both spontaneous and carcinogen-induced rat mammary tumors carry genetic mutations that are rarely seen in human BCs, such as *Ras* mutations, the other modeling strategies could be superior at recapitulating the disease [131, 132]. For instance, somatic mutations in radiation-induced tumors were found in signaling pathways also relevant to human breast cancer, [240, 242] whilst *Hras* and *Tp53* mutations were lacking [239]. Above all, the use of genetically engineered models enables precise, tissue-specific edits of (combinations of) driver genes of interest, thus ensuring an accurate genetic recapitulation of the human disease [21, 279, 280].

In addition to achieving a similar BC genetic makeup in the rat mammary tumors, other BC features could be recapitulated in vivo by combining the different models and exploiting their synergistic effects. Such experimental practice has previously been explored by Segaloff and Maxfield [316] and Broerse et al. [231], who investigated the combined effect of irradiation and estrogen supplementation on rat mammary tumorigenesis. In both studies, the additive effect of radiation and hormones was demonstrated, as seen by an increase in mammary carcinoma incidence in the presence of both agents [231, 316]. Furthermore, in the context of genetically modified models, previous studies in mice have highlighted a potential synergism between genetic engineering methods and induced hormonal disturbances, such as exogenous estrogen supplementation and ovariectomy [317, 318]. In particular, Dabrosin et al. reported an enhanced tumor growth rate in genetically engineered animals allografted with tumor cells and supplemented with estradiol [317]. Though performed in mouse models, a similar approach could be employed in rats with the goal of establishing the ideal in vivo combinatorial conditions to recapitulate the human disease.

Though these models are promising tools to study and more reliably recapitulate HR^+^ BC development and progression in vivo, they also display several limitations. As with most animal experimental models, animal welfare, ethical and legal concerns must be taken into account, as well as the need for skilled personnel, and adequate housing and husbandry conditions [319, 320]. Moreover, in vivo experiments are often labor-intensive, cost-inefficient and time-consuming, resulting in models with long tumor latencies. In the case of rats, another drawback concerns the limited availability of resources and tools that can be applied to the species. For instance, while over 120,000 disease-related functional annotations have been made on human and mouse genes, less than 11,000 annotations can be found for rat genes [321]. Similarly, recent pioneering efforts into annotating the rat transcriptome have yet to achieve the unmet level of detail inherent to mouse and human transcriptomic atlases [322–326]. Along these lines, even though the concept of intrinsic molecular subtypes has revolutionized the clinical understanding and management of BC, rat mammary tumors are thus far almost always simply described as HR-positive or -negative without mentioning their relevance to the intrinsic subtype. It would be of great importance to place the historically generated rat models in the context of these intrinsic subtypes to better stratify model utility and applicability.

Notwithstanding these limitations, rat models are valuable research tools for studying human malignancies, including HR^+^ BCs, and their further exploration will offer exciting opportunities for the disease modeling field. In addition to the rat mammary tumor models described thus far, novel modeling strategies to be explored include the engraftment of rat-derived tumor organoids into syngeneic rats, and the use of prime editing technology. Concerning the former, and though allograft models have previously been established using tumor pieces or cell lines, the development of rat HR^+^ organoid lines and their subsequent engraftment into syngeneic rats is still to be reported. Given that organoids can undergo genetic manipulation, including the introduction of reporter constructs and targeted genetic alterations, as well as fast expansion for drug screening prior to in vivo experimentation, their successful transplantation into rats could provide a new platform to model the disease. Due to the drastically divergent resources needed for experimental manipulation of in vitro and in vivo settings, organoid allograft modeling could represent a time- and cost-efficient alternative to studies entirely executed in vivo [188].

Future advances in genetic modeling in rats could also be achieved through the use of prime editing tools, such as the recently developed precise genome editing methods enabling both germline and somatic manipulation of cancer driver genes of interest with limited off-target effects [283, 327]. As seen in mouse models, prime editing enables successful introduction of a broad spectrum of cancer-associated mutations, including transversions, multi-nucleotide substitutions and deletions with over 90% editing purity.

In conclusion, rat models display a number of advantages for the study of human HR^+^ BCs, including the biological similarities to the human breast, the histopathological and morphological features displayed by the rat tumors, their ductal origin, and hormone dependency status [96, 99, 109, 146, 328]. The different rat models established to date display unique advantages and disadvantages, and enable a broad spectrum of different research applications. To further exploit the potential of rats in modeling HR^+^ BC, the different models and tumor induction methods could be employed in combination, with ample opportunity to propel pre-clinical HR^+^ BC research.

## Data Availability

No datasets were generated or analysed during the current study.
